# Predicting cardiovascular disease morbidity and mortality in chronic kidney disease in Spain. The rationale and design of NEFRONA: a prospective, multicenter, observational cohort study

**DOI:** 10.1186/1471-2369-11-14

**Published:** 2010-07-07

**Authors:** Mireia Junyent, Montserrat Martínez, Mercè Borràs, Blai Coll, Jose Manuel Valdivielso, Teresa Vidal, Felipe Sarró, Jordi Roig, Lourdes Craver, Elvira Fernández

**Affiliations:** 1 Nephrology Department, Institut de Recerca Biomèdica de Lleida at Hospital Universitari Arnau de Vilanova, Lleida, Spain

## Abstract

**Background:**

Cardiovascular disease (CVD) is the leading cause of morbidity and mortality in patients with chronic kidney disease (CKD). Cardiovascular risk assessment in this population is hampered by the failure of traditional risk factors to fully account for the elevated CVD risk (reverse epidemiology effect) and the presence of emerging risk factors specifically related to kidney failure. Therefore, diagnostic tools capable of improving cardiovascular risk assessment beyond traditional risk factors are currently warranted. We present the protocol of a 4-year prospective study aimed to assess the predictive value of non-invasive imaging techniques and biomarkers for CVD events and mortality in patients with CKD.

**Methods:**

From November 2009 to October 2010, 4137 asymptomatic adult patients with stages 2 to 5 CKD will be recruited from nephrology services and dialysis units throughout Spain. During the same period, 843 participants without CKD (control group) will be recruited from lists of primary care physicians, only at baseline. During the follow-up, CVD events and mortality will be recorded from all CKD patients. Clinical and laboratory characteristics will be collected in a medical documentation sheet. Three trained itinerant teams will carry out a carotid ultrasound to assess intima-media thickness and presence of plaques. A composite atherosclerosis score will be constructed based on carotid ultrasound data and measurement of ankle-brachial index. In CKD patients, presence and type of calcifications will be assessed in the wall of carotid, femoral and brachial arteries, and in cardiac valves, by ultrasound. From all participants, blood samples will be collected and stored in a biobank to study novel biomarkers.

**Conclusions:**

The NEFRONA study is the first large, prospective study to examine the predictive value of several non-invasive imaging techniques and novel biomarkers in CKD patients throughout Spain. Hereby, we present the protocol of this study aimed to explore the most effective way in which these tests can be integrated with traditional risk factors to maximize CVD detection in this population.

## Background

Cardiovascular disease (CVD) is the leading cause of cardiovascular morbidity and mortality in patients with chronic kidney disease (CKD) [1,2]. This association is even more evident in dialysis patients, where CV mortality accounts for 45% of all-cause mortality [3]. Therefore, as stated by the American Heart Association (AHA), CKD patients should be considered as CHD risk equivalent for risk factor management [4].

Multivariable mathematical functions based on traditional cardiovascular risk factors, such as the Framingham Risk Score charts [5], have been developed for predicting CVD risk in individual patients. However, these risk equations provide an inadequate warning to asymptomatic patients with CKD due to the reverse epidemiology effect [6], by which traditional risk factors fail to fully account for the elevated CVD risk in this population, and the presence of non-traditional biomarkers specifically related to kidney failure *per se *[7]. Moreover, recent studies suggest only limited utility of either single or multiple biomarkers of inflammation, oxidative stress, anemia, endothelial dysfunction, vascular calcification and electrolyte imbalance, as prognostic tools of patients with CKD [8]. Therefore, as discussed by the Screening for Heart Attack Prevention and Education (SHAPE) Task Force [9], additional tools capable of improving cardiovascular risk assessment beyond traditional risk factors are clearly warranted in this population.

Among these techniques, carotid ultrasound and ankle-brachial index (ABI) are non-invasive, simple, widely available, relatively inexpensive, and generally reproducible methods to assess carotid atherosclerosis and peripheral arterial disease (PAD), respectively [10,11]. Several prospective studies [12,13] in the general population have shown that an increased carotid intima-media thickness (CIMT) and/or abnormal ABI are associated with a higher CVD risk, independently of traditional risk factors. Prior studies have also examined the prognostic value of CIMT [14-18] or ABI [19,20] in CKD patients. However, most of them were conducted in patients on dialysis therapy, except by Szeto et al [18] who analyzed the predictive role of CIMT in a small sample of patients in predialysis. This group of investigators [18] also found that CIMT was a valuable tool for CVD risk stratification. Therefore, further studies are needed to ascertain the predictive role of these tests, particularly in early stages of CKD.

On the other hand, CKD patients have a higher prevalence of alterations in their calcium and phosphorus homeostasis that ultimately result in vascular calcifications. Presence of vascular calcifications in the arterial walls and cardiac valves has been associated with an increased risk for CV events and mortality [21,22]. However, the impact on prognosis according to the extent and type of calcification (intimal *vs. *medial) remains still unclear [23,24].

Therefore, the NEFRONA study is aimed to assess atherosclerosis burden and vascular calcifications in a large cohort of Spanish patients with CKD. This study will also examine the predictive value of non-invasive imaging techniques and novel biomarkers influencing cardiovascular events and mortality in all forms of CKD. Finally, the present study will allow us to assess whether these tests can be integrated with traditional risk factors to maximize early-detection of CVD in this population.

## Methods

### Study Design

In a multicenter, prospective (four years of follow-up) study, 4137 patients with CKD will be enrolled from November 2009 to October 2010. CKD patients will be recruited from different hospitals throughout Spain referred by their nephrologists. All patients will undergo: 1. ultrasound assessment of carotid, femoral and brachial arteries, 2. ABI, and 3. echocardiography. These tests will be carried out according to a common protocol by three trained itinerant teams composed each one of a technician and a nurse. During the same period, 843 patients without CKD (control group) will be recruited from lists of primary health physicians. The local Ethics Committee of the Hospital Arnau de Vilanova approved the protocol. All subjects will provide informed consent to participate in the study.

### Study participants

Participants will be eligible for inclusion if they (1) have CKD (glomerular filtration rate (GFR) < 60 ml/min per 1.73 m^2^) stages 2 to 5, and (2) ages of 18 to 74 years. Patients undergoing hemodialysis (HD) or peritoneal dialysis (PD) will be also invited to participate. The study is designed to use a population control as reference group. Eligible controls will be asymptomatic subjects without CKD. Participants will be excluded for any of the following reasons: (1) had prior CVD, (2) received a kidney transplant, (3) had a life-threatening disease, such as cancer, (4) underwent previous carotid artery surgery, or (5) have an ABI < 0.7 and/or a carotid artery stenosis greater than 75% at baseline.

We will use the Modification of Diet in Renal Disease Study (MDRD) and the Chronic Kidney disease Epidemiology Collaboration (CKD-EPI) equations to estimate the GFR to classify patients into the different stages of CKD [25,26]. Patients switching stage or dialytic modality in the follow-up of this study will be considered and these data will be registered at each visit. Only patients who switch to kidney transplant will be excluded from the follow-up. Enrollment will be stratified by hospital according to the prevalence of CKD patients in each stage, in order to obtain a sample in which all Spanish regions would be represented. Details about centers and geographical distribution can be seen at the website of the NEFRONA study http://www.nefrona.es.

### Clinical and laboratory characteristics

At each study visit, physicians responsible for the recruitment of participants will be asked to complete a medical questionnaire in patients with (Table 1) and without (Table 2) CKD. Briefly, these questionnaires will include data regarding family history of early-onset CVD, clinical history, cardiovascular risk factors, analytic values, and medication use. Information on smoking habit and alcohol intake (grams of alcohol) will be obtained by direct questioning. Anthropometrical data will be obtained by standard methods. Weight will be measured with a beam balance and height with fixed stadiometer. BMI will be calculated as weight, in kilograms, divided by the square of height, in meters. Waist circumference will be measured at the umbilicus, measured in meters. Blood pressure will be measured in duplicate with a validated semi-automatic oscillometer (Omron HEM-705CP) while patients are seated and had rested for five min. Hypertension will be defined as systolic blood pressure (BP) ≥140 mmHg, diastolic BP ≥90 mmHg, or current use of antihypertensive medication. Diabetes mellitus will be defined as a fasting glucose level ≥126 mg/dL (6.93 mmol/L) or treatment with antidiabetic agents. Analytic values will be obtained in fasting from recent clinical records.

**Table 1:** Questionnaire in patients with chronic kidney disease (CKD)

*INDIVIDUAL PATIENT DATA*			Date ____/____/________		
**Centre**:			**Identification number**:		
Sex:	men □	women □	**Age**:		
**Visit**:	VO V1 V2 V3 V4		**CKD stage**:	2 3 4 5	
**Glomerular Filtration Rate (CKD-EPI):**			**CKD etiology**:		

***DIALYSIS:***	Yes □	No □	Modality: HD □	PD □	Time on dialysis (months):
**HEMODIALYSIS (HD)**:			membrane type:	low flux □	high flux □
Frequency HD:	3 d/week □	/48 h □	/24 h □	Duration HD (hours):	
KtV (last measured value):					
Type of HD access:		arteriovenous shunt □		graft □	catheter □
**PERITONEAL DIALYSIS (PD)**:			Type:	Automatic □	Manual □
Transport type:	low □	high □	Icodextrin administration:	Yes □	No □
Total infused volume (ml):			Total eliminated volume (ml):		
diuresis volume (ml):			residual renal function (ml/min):		
Total KtV (last measured value):			peritoneal ktV (last measured value):		
Peritonitis (last year):	Yes □	No □	number of peritonitis:		

***COMORBIDITIES***					
Diabetes:			Yes □	No □	
Hypertension:			Yes □	No □	
Heart failure:			Yes □	No □	
Atrial fibrillation:			Yes □	No □	
Dyslipidemia:			Yes □	No □	
Family history of early cardiovascular disease:			Yes □	No □	
Parathyroidectomy:			Yes □	No □	

***LABORATORY DATA***			Date ____/____/________		
Hemoglobin (g/dl):			Urea (mg/dl):		
Hematocrit (%):			Uric acid (mg/dl):		
Iron (mg/dl):			Glucose (mg/dl):		
Transferrin (mg/dl):			HbA1c (%):		
Ferritin (ng/ml):			Insulin (U/l):		
Total calcium (mg/dl):			Cystatin C (mg/l):		
Phosphorus (mg/dl):			Total cholesterol (mg/dl):		
Intact parathormone level (pg/mL):			Triglycerides (mg/dl):		
Sodium (mEq/L):			HDL cholesterol (mg/dl):		
Potassium (mEq/L):			LDL cholesterol (mg/dl):		
Albumin (g/dl):			Hepatitis C virus positive:	Yes □	No □
Creatinin (mg/dl):			Microalbuminuria (mg/l) (urine):		
AST/ALT (U/L):			Albumin/creatinin (mg/g) (urine):		

***MEDICAL TREATMENT***			Platelet inhibitors:	Yes □	No □
Antihypertensives:	Yes □	No □	Oral anticoagulants:	Yes □	No □
Length of treatment (months):			Phosphate binders:	CO_3_Ca □	sevelamer □
Angiotensio-converting enzyme (ACE)			calcium acetate □	Al(OH)_3 _□	lanthane □
inhibitors:	Yes □	No □	Total dose (g/day):		
Angiotensin II	receptor	blockers	Lenght of treatment (months):		
(ARB):	Yes □	No □	Vitamin D analogues/metabolites:	Yes □	No □
Hypolipidemics:	statins □	fibrates □	Type:		
Length of treatment (months):			Total dose (mcg/week):		
Insulin:	Yes □	No □	Length of treatment (months):		
Oral antidiabetics:	Yes □	No □	Erythropoiesis-stimulating agents:	Yes □	No □

**Table 2:** Questionnaire in subjects with normal renal function (control group)

*INDIVIDUAL PATIENT DATA*					
**Centre**:			**Identification number**:		
Sex:	men □	women □	Age:		
***COMORBIDITIES***					
Diabetes:			Yes □	No □	
Hypertension:			Yes □	No □	
Heart failure:			Yes □	No □	
Atrial fibrillation:			Yes □	No □	
Dyslipidemia:			Yes □	No □	
Family history of early cardiovascular disease:			Yes □	No □	
Parathyroidectomy			Yes □	No □	

***LABORATORY DATA***			Date ____/____/________		
Hemoglobin (g/dl):			Urea (mg/dl):		
Hematocrit (%):			Uric acid (mg/dl):		
Iron (mg/dl):			Glucose (mg/dl):		
Transferrin (mg/dl):			HbA1c (%):		
Ferritin (ng/ml):			Insulin (U/l):		
Total calcium (mg/dl):			Cystatin C (mg/l):		
Phosphorus (mg/dl):			Total cholesterol (mg/dl):		
Intact parathormone level (pg/mL):			Triglycerides (mg/dl):		
Sodium (mEq/L):			HDL cholesterol (mg/dl):		
Potassium (mEq/L):			LDL cholesterol (mg/dl):		
Albumin (g/dl):			Hepatitis C virus positive:	Yes □	No □
Creatinin (mg/dl):			Microalbuminuria (mg/l) (urine):		
AST/ALT (U/L):			Albumin/creatinin (mg/g) (urine):		

***MEDICAL TREATMENT***					
Antihypertensives:			Yes □	No □	
Length of treatment (months):					
Angiotensio-converting enzyme (ACE) inhibitors:			Yes □	No □	
Angiotensin II Receptor Blockers (ARB):			Yes □	No □	
Hypolipidemics:	statins □	fibrates □	Length of treatment (months):		
Insulin:	Yes □	No □	Oral antidiabetics:	Yes □	No □
Platelet inhibitors:	Yes □	No □	Oral anticoagulants:	Yes □	No □

### Clinical follow-up and end-points

All CKD patients will be monitored each year for 4 years or until death, disenrollment from the hospital, or kidney transplantation. The clinical management will be decided by individual physician without being affected by the study. Primary end-points will be CVD events according to the International Classification of Diseases, Ninth Revision, Clinical Modification (ICD9-CM) which includes unstable angina, myocardial infarction, transient ischemic attack, cerebrovascular accident, congestive heart failure, arrhythmia, PAD or amputation for vascular disease and aorta aneurism. At each visit, all-cause and cardiovascular mortality will be also registered. All-cause mortality will comprise infections, tumors, accidents, and uremia. Cardiovascular mortality causes will include myocardial infarction, arrhythmia, congestive heart failure, stroke, aneurism, mesenteric infarction, and sudden death. Importantly, CVD events and deaths will be accurately recorded by each physician responsible of the recruitment of CKD patients. In the case of an out-of-hospital death, family members will be interview by telephone to better ascertain the circumstances surrounding death.

Secondary end-points will be CIMT and plaque presence, by ultrasound; ABI measurement; presence of calcifications in carotid, femoral, and brachial arteries and in cardiac valves, by ultrasound; left ventricular (LV) hypertrophy, LV ejection fraction, left atrial size, and LV diastolic filling, by echocardiography; and biomarkers. These tests will be performed during a midweek dialysis day in HD patients and on a full abdomen in PD patients.

### Carotid ultrasound

B-mode ultrasound imaging of the right and left carotid arteries will be performed using a General Electric instrument (Vivid i BT09 model) equipped with 6-13 MHz broadband linear array transducers. Participants will be examined in the supine position with the head turned 45°contralateral to the side of scanning. Prior to obtaining images for CIMT measurements, B-mode and color Doppler sonographic examinations will be done in longitudinal and transverse planes to identify vascular stenosis. A standardized imaging protocol will be use for CIMT measurements. With the carotid dilatation and flow dividers as anatomic landmarks, the sonographer will obtain high-resolution images of the common carotid (1 cm proximal to the bifurcation), the bifurcation (between dilatation and flow divider) and the internal carotid (1 cm distal to the flow divider). A single lateral angle of insonation, optimizing the image for the arterial far wall, will be use. Plaques will be sought by using B-mode and color Doppler examinations in both longitudinal and transverse planes to take into consideration circumferential asymmetry and will be defined as focal intrusions into the lumen ≥1.2 mm thick. We will consider severe stenosis if systolic velocity peak is higher or equal than 125 cm/s. We will define the following variables: 1. mean CIMT, defined as the average of 4 to 8 distances between the far wall lumen-intima and media-adventicia ultrasound interfaces taken bilaterally in each of the specified segments mentioned above, and 2. maximum CIMT, defined as the maximum distance in each segment. CIMT measurements will be made in plaque-free arterial segments. Digital images from diastolic frame recordings will be electronically transferred to an offline workstation (Hospital Arnau de Vilanova, Lleida, Spain) for quantification of CIMT measurements. Three certified ultrasound readers, unaware of patient clinical information, will review the scans and will made CIMT quantitative measurements, using a semi-automated edge-tracking software (SonoCalc IMT^®^) based on detection of the echo structures, with the option of making manual corrections.

### Ankle-brachial index (ABI)

From all participants, ABI will be obtained using a vascular Doppler MD2 Hungleight with an 8 MHz transducer. ABI is defined as the ratio of the systolic BP at the ankle to the systolic BP in the arm. A hand-held Doppler will be used to assess bilateral brachial, tibial and dorsalis pedis arteries. At each ankle (right and left), the higher of the two pressures measured in each leg will be used as the numerator for same-side ABI calculation. We will record ABI as the lowest value obtained at each territory.

### Atherosclerosis score

**Figure 1:**
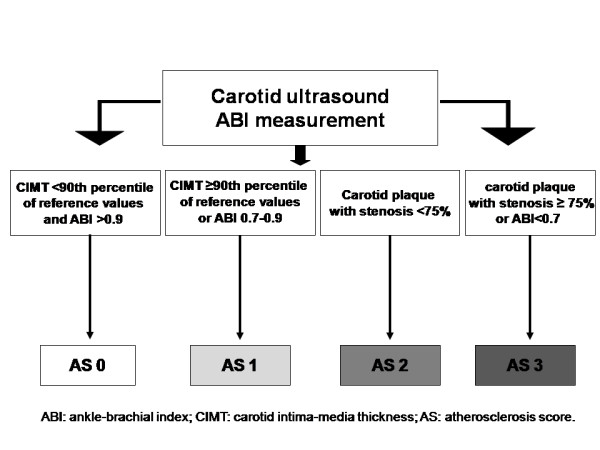
**Atherosclerosis score (AS) distribution depending on carotid ultrasound data and ankle-brachial index (ABI)**.

Based on carotid ultrasound data and ABI measurement, we will set up an atherosclerosis score (AS) defined as follows (Figure 1):

1. No atherosclerosis (AS 0): ABI >0.9 and CIMT values <90th percentile of reference values [26].

2. Mild atherosclerosis (AS 1): an ABI between 0.7-0.9 or CIMT values ≥90th percentile of reference values.

3. Moderate atherosclerosis (AS 2): carotid plaque with stenosis <75%.

4. Severe atherosclerosis (AS 3): an ABI <0.7 or carotid plaque with stenosis ≥75%.

### Vascular calcifications

In CKD patients, vascular calcifications will be assessed by B-mode ultrasound in both sides of carotid, femoral, and brachial arteries. Carotid calcifications will be assessed in each of the specified carotid segments mentioned above, whereas femoral calcifications will be examined in common and superficial femoral arteries. Highly echogenic plaques producing bright white echoes with shadowing will be considered to be calcifications. Arterial calcifications at each arterial site will be quantified qualitatively as absent (0) or present (1). The final score will be obtained by the addition of calcifications from all studied territories, ranging from 0 (absence of calcium deposit) to 12 (calcifications present in all territories examined). Type of calcifications will be also registered according to their location in the arterial wall, as follows: intimal, medial or both (plaque presence). Three certified ultrasound readers, unaware of patient clinical information, will review the scans and will determine the calcification score.

### Echocardiography

A standard two-dimensional M-mode color Doppler echocardiography will be performed on CKD patients using a 3 MHz transducer (General Electric Vivid i BT09). LV mass will be calculated as follows: 0.8 × [1.04 × (PWT + IVST + LVEDD)^3 ^- LVEDD^3^] - 13.6, where PWT is the posterior wall thickness, IVST is the interventricular septal thickness and LVEDD is the LV end-diastolic diameter. LV mass will be normalized to body height as an index in g/m^2^. LV hypertrophy will be defined according to Framingham's criteria (LV mass/body surface area [BSA] >110 g/m^2 ^in females and LV mass/BSA >134 g/m^2 ^in males) [28]. LV ejection fraction and left atrial size will be also measured. LV diastolic filling will be evaluated from pulsed Doppler studies obtained from the apical four-chamber view of the heart. Maximal early diastolic flow velocity (E) and maximal late atrium flow velocity (A) will be measured and their ratio (E/A) calculated. Presence of calcifications in mitral and aortic valves (yes/no) will be also evaluated. All images will be sent to one study site (Hospital Clínico San Carlos, Madrid, Spain) for analysis by two certified readers blinded to clinical data.

### Biomarkers

Samples will be collected of whole blood into 10 ml EDTA vacutainers, maintained at ambient temperature and transported overnight to a centralized biobank named REDinREN (Spanish Renal Research Network). These samples will be processed immediately after their reception by a laboratory technique according to the biobank protocol [29]. Plasma, serum, DNA and RNA will be collected from all participants. These samples will be frozen and stored in the biobank until their use.

### Sample size calculation

The sample size needed to address the objectives of this study has been estimated with the following assumptions: alpha 5%, beta 10% (power 90%), loss to follow-up 25%, and revised incidence of CV outcomes at each CKD stage [2]. Based on these data, the target number for inclusion of patients with CKD will be 4137, distributed for each stage as follows: 1476 (stage 2), 1325 (stage 3), 713 (stages 4 and 5), and 623 (patients in dialysis treatment, either HD or PD). The sample size of the control group will be 843 based on minimal detectable differences in plaque presence between patients with and without CKD (personal data from Hospital Arnau de Vilanova).

### Statistical Analyses

SPSS software (version 17.0) will be used for statistical analyses. Data will be presented as means ± SE for continuous variables and as frequencies or percentages for categorical variables. Differences in mean values will be assessed by analysis of variance and unpaired t-tests, or the Mann Whitney U-test when the variables have a skewed distribution. Categorical variables will be compared by using the Pearson chi-square or the Fisher's exact tests. The relationship between measured primary and secondary end-points will be analyzed by multivariate Cox regression. Survival curves will be constructed using the Kaplan-Meier method, and long-rank test will be used for exploratory univariate analysis of survival differences between groups. We will use ROC curves analysis to calculate the additional predictive value for CV outcomes of imaging parameters and biomarkers beyond and above that provided by traditional risk factors. Two-sided *P *values <0.05 will be considered statistically significant.

## Discussion

The present study is the first large, prospective, observational study to examine the usefulness of different non-invasive imaging techniques and biomarkers to predict CVD events and mortality in CKD patients throughout Spain. Given the paradoxical associations between traditional risk factors and CVD risk in patients with CKD, additional diagnostic tools are currently warranted to identify the real cardiovascular culprits. Hereby, we present the protocol of the NEFRONA study aimed to explore the most effective way in which these tests can be integrated with current clinical laboratory markers to maximize their synergism for CVD detection.

CIMT is a well-documented surrogate marker of preclinical atherosclerosis, by which higher CIMT measurements have been associated with an increased risk of CVD [11]. Several prior studies have assessed its predictive role in healthy subjects [12] and CKD patients [14-18]. However, most studies in CKD patients have been performed in small samples of patients undergoing dialysis therapy. Only Szeto et al [18] assessed CIMT in a sample of 203 Chinese patients on predialysis. The authors concluded that CIMT was a strong predictor of CVD and might be usefully applied for risk stratification in this group of patients. ABI as a surrogate marker to predict PAD, has also been assessed as a predictor of CVD mortality only in dialysis patients [19,20]. Therefore, the predictive value of either CIMT or ABI in early stages of CKD remains still unknown.

Vascular calcification is a common complication in CKD, and several investigators have demonstrated that presence and extent of vascular calcifications is an independent predictor of CV mortality [21-23]. Interestingly, Blacher et al [23] studied the prognostic value of calcifications in carotid artery, abdominal aorta, iliofemoral axis, and legs in 110 patients on HD therapy, reporting an increased risk of death in those patients with a higher number of calcified vascular sites. However, only one study [24] has examined the predictive value of type of arterial calcifications (intimal *vs. *medial) in 202 HD patients using radiographs of the pelvis and the thigh. London et al [24] found that intimal calcification was associated with atherosclerotic complications whereas medial calcification, with CKD specific risk factors. Moreover, the authors reported higher rates of CVD mortality in patients with intimal calcification. Further studies are currently warranted in order to clarify the importance of extent and type of vascular calcifications in different arterial territories and in large samples of patients with all sorts of CKD.

Calcifications of cardiac valves have also been associated with an increased risk for mortality and CVD events in healthy populations [21]. In CKD patients, only two prior small studies have examined the predictive role of cardiac valve calcifications with controversial results [30,31]. In this regard, Wang et al [30] reported that presence of calcified cardiac valves was predictive of all-cause mortality and cardiovascular death in 192 patients on dialysis. By contrast, Panuccio et al [31] did not find an association between cardiac valve calcifications and cardiovascular mortality in 202 HD patients. In addition, prior small studies have identified a number of echocardiographic variables, including left atrial size, LV hypertrophy, and LV filling pressure, that predict CVD events and mortality in dialysis patients [32,33]. However, no prospective studies have assessed their predictive value in predialysis patients. Thus, the NEFRONA study will provide us compelling evidence regarding the prognostic role of cardiac valve calcifications and several ecocardiographic variables in CKD patients. Moreover, this study may help us to understand the link between calcifications in the arterial walls or cardiac valves and atherosclerosis, a question that to date remains undetermined.

On the other hand, the collection of plasma, DNA and RNA samples in this study will contribute to a better assessment of CVD in renal patients. Although recent studies [8] suggest only limited utility of either single or multiple biomarkers of CVD risk as prognostic tools in CKD patients, their integration with traditional markers to maximize CVD detection in this population remains to be established. Of note, identification of specific genetic variants acting in the arterial walls will allow us to develop genetic screening tools for diagnosis and therapeutic interventions. Likewise, RNA samples will enable us to understand the molecular mechanisms leading to atherosclerosis in these patients, contributing to the reduction of their morbimortality risk due to CVD.

Overall, the NEFRONA study may provide new insights into the natural course, genotype-phenotype interactions and pathophysiology of CKD, providing new targets for early diagnosis and therapy for CVD in these patients. The present study has several strengths, including the large sample size nationally representative, the complete nature of the dataset, the existence of a central biobank, the use of clinically important primary end-points (CV morbidity and all-cause and CV mortality), the performance of all imaging techniques by three trained itinerant teams using a common protocol, and the large follow-up period of this study (4 years). Importantly, the longitudinal nature of the present study will allow us to determine the temporal and causal relationships among CVD risk factors, CKD, imaging parameters, and biomarkers. However, there are some limitations to our study. First, estimation of kidney function by determination of serum creatinine may be responsible for misclassification of some participants in the analyses by stage of kidney function; however, its determination will be made using the Jaffe kinetic method by all participating centers. Second, we will use estimated GFR rather than more precise measures of kidney function, like iothalamate clearance. Finally, prevalent dialysis patients will be included, which may introduce survival bias because of early mortality.

## Conclusions

In conclusion, the NEFRONA study will examine whether non-invasive imaging techniques and biomarkers may increase the predictive power to detect CV morbidity and mortality in asymptomatic CKD patients, beyond traditional risk factors. Moreover, the present study may enable us to understand the molecular mechanisms leading to atherosclerosis in these patients, contributing to the reduction of their morbimortality risk due to CVD.

## List of abbreviations used

(CVD): Cardiovascular disease; (CKD): chronic kidney disease; (AHA): American Heart Association; (SHAPE): Screening for Heart Attack Prevention and Education; (ABI): ankle-brachial index; (PAD): peripheral arterial disease; (CIMT): carotid intima-media thickness; (GFR): glomerular filtration rate; (HD): hemodialysis; (PD): peritoneal dialysis; (MDRD): Modification of Diet in Renal Disease Study; (CKD-EPI): Chronic Kidney disease Epidemiology Collaboration; (BP): blood pressure; (ICD9-CM): International Classification of Diseases, Ninth Revision, Clinical Modification; (LV): left ventricular; (AS): atherosclerosis score.

## Competing interests

The authors declare that they have no competing interests.

## Authors' contributions

EF: Principal Investigator; the conception and design of the study; MJ and EF: the writing of the manuscript draft; MJ, TV, MM, BC, JV, MB, EF: participated in design and co-ordination; helped to draft manuscript; read and approved the final manuscript; MJ, TV, MM, BC, JV, JR, FS, MB, EF: critical review of the manuscript; MJ, MM: statistical expertise; EF, MJ, BC, JV: obtained funding.

## Pre-publication history

The pre-publication history for this paper can be accessed here:

http://www.biomedcentral.com/1471-2369/11/14/prepub
